# *Azotobacter vinelandii* as a Nitrogen‐Negative Chassis for Bio‐Oil and Bio‐Wax Production of Heterologous and Native Lipids

**DOI:** 10.1002/mbo3.70047

**Published:** 2025-08-17

**Authors:** Brett M. Barney, Bilge Bahar Camur, Lucas J. Stolp, Natalia Calixto Mancipe, Benjamin R. Dietz

**Affiliations:** ^1^ Department of Bioproducts and Biosystems Engineering University of Minnesota St. Paul Minnesota USA; ^2^ Biotechnology Institute University of Minnesota St. Paul Minnesota USA

**Keywords:** *Azotobacter vinelandii*, fatty alcohols, neutral lipids, resorcinol, wax esters

## Abstract

The biosynthetic production of energy‐dense petrochemical substitutes is an important goal to address sustainability. The diazotrophic soil microbe *Azotobacter vinelandii* is a model microbe for the study of biological nitrogen fixation. In addition to capturing atmospheric nitrogen and converting it into usable nitrogen compounds, it is also regarded for the ability to accumulate the bioplastic poly‐β‐hydroxybutyrate and the extracellular polysaccharide alginate. Here, we demonstrate the potential to broaden the chemical products repertoire of *A. vinelandii* by demonstrating the accumulation of several classes of biological lipids and waxes. These products include the expanded accumulation of wax esters and fatty alcohols through heterologous expression of foreign genes and pathways, and increased production of the native lipid alkylresorcinol, accomplished by deregulating specific internal pathways and removing competitive pathways for alternative products. As a result, we demonstrate a sevenfold increase in the accumulation of alkylresorcinol, manifesting as intracellular inclusions that are easily extracted with simple solvents and account for nearly 20% of the cellular biomass. By selecting a diazotrophic microbe as a chassis for lipid accumulation, we produced these lipids without any requirement for industrial nitrogen sources in the growth medium, resulting in a net positive nitrogen process as well.

## Graphical abstract

Strains of the nitrogen‐fixing microbe *Azotobacter vinelandii* were restructured to remove competing metabolites, resulting in the hyper accumulation of the native lipid alkylresorcinol, visible as large inclusions within the cell.

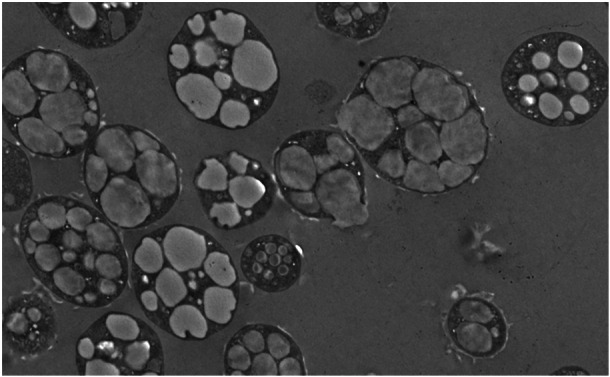

## 1 Introduction

The microbial production of petroleum alternatives through biosynthetic routes represents an important pillar to meet the energy and industrial needs of a growing and sustainable society. Many of the current biofuels markets, such as bioethanol from corn (Pereira et al. 2019), biodiesel from plant oilseeds (Hill et al. 2006), algal biofuels (Pienkos and Darzins 2009), or methane production from anaerobic digestion, may contribute to circular carbon markets, but still require high inputs of nitrogen derived from industrial processes, such as Haber Bosch (Erisman et al. 2008; Smil 1999). These industrial processes require a significant expenditure of energy to fix atmospheric nitrogen to grow or maintain the initial biomass sources. Additionally, many industrial fermentations and alternative biosynthetic routes utilize common bacterial and yeast strains that also require a nitrogen input to grow their biomass (Steen et al. 2010; Stöveken and Steinbüchel 2008).

The soil microbe *Azotobacter vinelandii* has a long history of research interest as a model microbial system for the study of biological nitrogen fixation (Barney 2024; Jensen 1954; Noar and Bruno‐Bárcena 2018). Among nitrogen‐fixing microbes (diazotrophs), it has some novelty related to its ability to fix nitrogen during aerobic growth, even though the nitrogenase enzyme responsible for fixing nitrogen is extremely sensitive to oxygen, which destroys the complex metal cofactors involved in catalysis (Burén et al. 2020). *A. vinelandii* has been studied for more than 120 years in relation to nitrogen fixation (Barney 2024; Barney et al. 2006), accumulation of the bioplastic poly‐β‐hydroxybutyrate (PHB) (García et al. 2014), production of the extracellular carbohydrate alginate (Sabra et al. 2000; Peña et al. 2002), production of metal‐mining siderophores (Fekete et al. 1983; Villa et al. 2014), resistance to desiccation through the formation of cysts (Stevenson and Socolofsky 1966; Segura, Cruz, et al. 2003), and potential to function in aquaculture and agricultural settings as a biofertilizer (Ortiz‐Marquez et al. 2012; Barney, Eberhart, et al. 2015), among its many other interesting features. In contrast, it is not generally regarded for its potential to serve as a chassis for biofuel production.

Many bacteria accumulate PHB as a form of carbon and energy storage during growth on a supply of unbalanced nutrients (excess carbon in comparison to nitrogen or other limiting nutrients, Wältermann et al. 2005). The accumulation of neutral lipids is less prominent in bacteria, and is generally associated with higher land plants, animals, and microalgae. However, certain bacteria, including *Acinetobacter*, *Rhodococcus*, and *Marinobacter,* are known to accumulate triacylglycerides, wax esters or a combination of both (Wältermann et al. 2005, 2007; Barney et al. 2012). *A. vinelandii* does not produce triacylglycerides or wax esters, but does accumulate small quantities of an alternative biological wax called alkylresorcinol as a component of the membranes during cyst formation (Segura, Cruz, et al. 2003; Funa et al. 2006). Alkylresorcinols (resorcinolic lipids) are natural waxes that are energy‐rich hydrocarbons that replace native membrane lipids during the transition of cells to the dormant cyst state. In addition to *A. vinelandii*, they are also found in many higher land plants, where they have been proposed to protect the plants from certain pathogenic fungi, and may play a role in animal health as well (Zabolotneva et al. 2022). These energy‐rich hydrocarbons are easily extracted from dry cell mass using simple solvents and can be further processed or cracked using petrochemical processes to produce a range of fuels and oils.

In this study, we evaluated the potential to produce wax esters and fatty alcohols using nonnative enzymes in *A. vinelandii* as a biological chassis. Importantly, lipids were produced using a simple medium of sucrose devoid of any reduced nitrogen compounds, and obtained all of the nitrogen required for growth through biological nitrogen fixation, making the process nitrogen negative. In the process of strain optimization, we were also able to substantially increase the levels of the native lipid alkylresorcinol to levels almost sevenfold higher than what was obtained from the parent DJ (laboratory wild‐type) strain, resulting in the accumulation of these lipids in the cytoplasm as intracellular inclusions that were easily extractable.

## 2 Materials and Methods

### 2.1 Materials

*A. vinelandii* strain DJ (ATCC BAA‐1303) was kindly provided by Dennis Dean. *Acinetobacter baylyi* (ATCC 33305) and *Marinobacter aquaeolei* VT8 (ATCC 700491) were obtained from the American Type Culture Collection. *Escherichia coli* JM109 was obtained from New England Biolabs (Ipswich, MA). Silica gel (60 Å), dichloromethane, *n*‐hexane, diisopropyl ether, acetic acid, and tetrahydrofuran were american chemical society grade or better and purchased from Sigma‐Aldrich (St. Louis, MO). All other chemicals and reagents used in this study were obtained from Fisher Scientific (Pittsburgh, PA) or Sigma‐Aldrich Chemical Company.

### 2.2 Culture Growth

*A. vinelandii* was grown in Burk's medium at 26°C on a shaker table at 180 rpm (Dos Santos 2011; Plunkett et al. 2020).

### 2.3 Strain Construction

All strains were constructed following methods that have been described elsewhere (Dos Santos 2011; Dietz et al. 2024; Eberhart et al. 2016). For methods that used counterselection and SacB toxicity, strains containing the *sacB* gene were first grown on Burk's medium with glucose substituted for sucrose. A list of strains and genotypes used in this study is provided in Table 1. A list of plasmids and their characteristics is provided in Table 2, and the primers used to clone genes and move them into these plasmids are provided in Table 3.

**Table 1:** Mutant strains constructed and/or used in this study.

*Azotobacter vinelandii* strain	Plasmid utilized	Genetic features	Parent strain/reference
DJ	None	Wild‐type	
AZBB072		Δ*pyrF*	Eberhart et al. (2016)
AZBB131		Δ*phbBAC*	Eberhart et al. (2016)
AZBB517	pPCRTAGD4	Δ*Avin_06840*::*pyrF*‐kan^R^, Δ*pyrF*	AZBB072
AZBB520	pPCRTAGD2	Δ*Avin_06840*, Δ*pyrF*	AZBB517
AZBB539	pPCRARS63	Δ*arsABCD*::*pyrF*‐kan^R^, Δ*Avin_06840*, Δ*pyrF*	AZBB520
AZBB560	pPCRARS62‐2	Δ*arsABCD*, Δ*Avin_06840*, Δ*pyrF*	AZBB539
AZBB586	pPCRALGD6	*algD.8.44.KJGXLIVFA*::*pyrF*‐kan^R^, Δ*pyrF*	AZBB072
AZBB589	pPCRDPHB4	Δ*Avin_34710*::*pyrF*‐kan^R^, Δ*arsABCD*, Δ*Avin_06840*, Δ*pyrF*	AZBB560
AZBB638	pPCRDPHB2	Δ*Avin_34710*, Δ*arsABCD*, Δ*Avin_06840*, Δ*pyrF*	AZBB589
AZBB639	pPCRALGD5	Δ*algD.8.44.KJGXLIVFA*, Δ*pyrF*	AZBB586
AZBB652	pPCRPYRF1	Δ*algD.8.44.KJGXLIVFA*	AZBB639
AZBB653	pPCRPYRF1	Δ*Avin_34710*, Δ*arsABCD*, Δ*Avin_06840*	AZBB638
AZBB682	pPCRALC83‐3	*phbB*::*ACIAD0832‐Maqu_2220*‐kan^R^, Δ*Avin_34710*, Δ*arsABCD*, Δ*Avin_06840*	AZBB653
AZBB712	pPCRALGD11	*algD.8.44.KJGXLIVFA*::*lacZ*‐tet^R^, Δ*phbBAC*	AZBB131
AZBB719	pPCRALGD12	*algD.8.44.KJGXLIVFA*::*lacZ*‐tet^R^ with single homologous recombination of pPCRALGD12, Δ*phbBAC*	AZBB712
AZBB721		Δ*algD.8.44.KJGXLIVFA*, Δ*phbBAC*	AZBB719
AZBB744	pPCRALC88	*Avin_16040*::*ACIAD0832‐Maqu_2220*‐strep^R^, Δ*algD.8.44.KJGXLIVFA*, Δ*phbBAC*	AZBB721
AZBB805	pPCRFAD7	*Avin_15490*::tet^R^, *Avin_16040*::*ACIAD0832‐Maqu_2220*‐strep^R^, Δ*algD.8.44.KJGXLIVFA*, Δ*phbBAC*	AZBB744
AZBB808	pPCRFAD7	*Avin_15490*::tet^R^, Δ*algD.8.44.KJGXLIVFA*, Δ*phbBAC*	AZBB721
AZBB821	pPCRRSMA3	*Avin_34440*(*rsmA*)::tet^R^, Δ*algD.8.44.KJGXLIVFA*, Δ*phbBAC*	AZBB721
AZBB822	pPCRALC73	*Avin_16040*::*Maqu_2220*‐strep^R^, *Avin_15490*::tet^R^, Δ*algD.8.44.KJGXLIVFA*, Δ*phbBAC*	AZBB808

**Table 2:** Key plasmids and relevant derivatives of these plasmids used for the construction of *Azotobacter vinelandii* manipulated strains.

Plasmid^a^	Relevant genes cloned or plasmid manipulation	Parent vector(s)	Reference and/or source
pBB053	pUC19 derivative.		Lenneman et al. (2013)
pBB114	pUC19 derivative with kanamycin resistance in place of ampicillin resistance.		Lenneman et al. (2013)
pBB284	BBa_J72214‐BBa_J72090 segment containing deoxyviolacein cassette in pUC19 derivative backbone.	pBB053	Barney and Dietz (2024)
pBBTET3	pUC19 derivative with tetracycline resistance in place of ampicillin resistance.		Barney and Plunkett (2022)
pBBTET6	Plasmid containing a tetracycline resistance cassette.		Schwister et al. (2022)
pLACZF19	Plasmid containing *lacZ* and tetracycline cassette.		Dietz et al. (2024)
pPCRALC73	Moved Maqu_2220 gene from pPCRALC29 into pPCRUNKK31 to express Maqu_2220 behind Avin_16040 gene promoter with streptomycin selection.	pPCRALC29, pPCRUNKK37	Barney and Plunkett (2022) and Wahlen et al. (2009)
pPCRALC78	Moved the Maqu_2220 gene behind the ACIAD0832 gene in pPCRWE391.	pPCRWE391, pPCRALC73	This study
pPCRALC83‐3	Fused ACIAD0832 and Maqu_2220 genes into a single gene product separated by seven asparagine residues.	pPCRALC78	This study
pPCRALC88	Moved fused ACIAD0832/Maqu_2220 gene behind Avin_16040 gene promoter with streptomycin selection.	pPCRALC83‐3, pPCRUNKK40	This study
pPCRALGD3	Cloned the flanking region downstream of *algA* into pBB114.	pBB114	This study
pPCRALGD4	Cloned the flanking region upstream of *algD* into pBB284.	pBB284	This study
pPCRALGD5	Combined the flanking regions upstream of *algD* and the flanking region downstream of *algA* into a single plasmid.	pPCRALGD3, pPCRALGD4	This study
pPCRALGD6	Inserted kanamycin and *pyrF* gene cassette from pPCRKAN15 between flanking regions of pPCRALGD5 for removal of *algD.8.44.KJGXLIVFA* operon.	pPCRALGD5, pPCRKAN15	This study
pPCRALGD11	Inserted tetracycline and *lacZ* gene cassette from pLACZF19 between flanking regions of pPCRALGD5 for removal of *algD.8.44.KJGXLIVFA* operon.	pPCRALGD5, pLACZF19	This study
pPCRALGD12	Inserted tetracycline and *lacZ* gene cassette from pLACZF19 between flanking regions of pPCRALGD5 for removal of *algD.8.44.KJGXLIVFA* operon.	pPCRALGD5, pLACZF19	This study
pPCRARS60	Cloned the flanking region upstream of *arsA* into pBB284.	pBB284	This study
pPCRARS61	Cloned flanking downstream of *arsD* into pBB114.	pBB114	This study
pPCRARS62‐2	Combined the flanking regions upstream of *arsA* and the flanking region downstream of *arsD* into a single plasmid.	pPCRARS60, pPCRARS61	This study
pPCRARS63	Inserted kanamycin and *pyrF* gene cassette from pPCRKAN15 between flanking regions of pPCRARS62‐2 for removal of *arsABCD* operon.	pPCRARS62‐2	This study
pPCRDPHB1	Cloned the Avin_34710 gene and flanking region into pBB053.	pBB053	This study
pPCRDPHB2	Removed Avin_34710 gene from pPCRDPHB1, leaving flanking regions behind.	pPCRDPHB1	This study
pPCRDPHB4	Inserted the kanamycin and *pyrF* gene cassette from pPCRKAN15 between the flanking regions of pPCRDPHB2.	pPCRDPHB2, pPCRKAN15	This study
pPCRFAD1	Cloned Avin_15490 gene and flanking region into pBB053.	pBB284	This study
pPCRFAD4	Removed Avin_15490 gene from pPCRFAD1, leaving flanking regions behind.	pPCRFAD1	This study
pPCRFAD7	Inserted tetracycline gene cassette from pBBTET6 between flanking regions of pPCRFAD4.	pPCRFAD4, pBBTET6	This study
pPCRKAN4	Plasmid containing a kanamycin selection marker cassette.		Barney, Eberhart, et al. (2015)
pPCRKAN15	Plasmid containing *the pyrF* gene and the kanamycin selection marker cassette.		Eberhart et al. (2016)
pPCRPHB40	Cloned the *phbB* gene and flanking region into pBB053.	pBB053	This study
pPCRPHB42	Removed *phbB* gene from pPCRPHB40 and incorporated multiple restriction enzyme sites to construct an expression vector.	pPCRPHB40	This study
pPCRPHB44‐2	Moved the kanamycin cassette from pPCRKAN4 into pPCRPHB42.	pPCRPHB42, pPCRKAN4	This study
pPCRPHB47	Optimized plasmid to express genes behind the *phbB* promoter in *A. vinelandii* with kanamycin selection.	pPCRPHB44‐2	This study
pPCRPRP7	Plasmid containing deoxyviolacein operon cassette.		Barney and Dietz (2024)
pPCRPYRF1	Plasmid containing the *pyrF* gene and flanking regions.		Eberhart et al. (2016)
pPCRRSMA1	Cloned Avin_34440 *rsmA* gene and flanking region into pBB284.	pBB284	This study
pPCRRSMA2‐2	Removed Avin_34440 gene from pPCRRSMA1, leaving flanking regions behind.	pPCRRSMA1	This study
pPCRRSMA3	Inserted tetracycline gene cassette from pBBTET6 between flanking regions of pPCRRSMA2‐2.	pPCRRSMA2‐2, pBBTET6	This study
pPCRSACB31	Restriction site optimized plasmid containing *sacB* and kanamycin resistance cassette.	pPCRSACB28	Dietz et al. (2024)
pPCRTAGD1	Cloned the Avin_06840 gene and flanking region into pBB053	pBB053	This study
pPCRTAGD2	Removed Avin_06840 gene from pPCRTAGD1, leaving flanking regions behind.	pPCRTAGD1	This study
pPCRTAGD4	Inserted the kanamycin and *pyrF* gene cassette from pPCRKAN15 between the flanking regions of pPCRTAGD2.	pPCRTAGD2, pPCRKAN15	This study
pPCRUNKK37	Gene expression vector to insert genes in place of Avin_16040 in *A. vinelandii* with streptomycin selection.		Barney and Plunkett (2022)
pPCRUNKK40	Optimized version of pPCRUNKK37 with additional restriction sites and deoxyviolacein operon for purple/white selection.	pPCRUNKK37	This study
pPCRWE390	Cloned the ACIAD0832 gene into the pBBTET3 plasmid.	pBBTET3	This study
pPCRWE391	Moved the ACIAD0832 gene into the pPCRPHB47 plasmid for expression of the gene behind the *phbB* promoter.	pPCRWE390, pPCRPHB47	This study

^a^ Sequences for all plasmids listed are available upon request.

**Table 3:** Primers used for the construction of *Azotobacter vinelandii* manipulated strains.

Primer	Sequence (all in 5′–3′ direction)	Plasmid construct
BBP2332	NNNGAATTCG ATTCTTGACA CGACATCCTT CTATATCATT G	pPCRPHB40
BBP2333	NNNAAGCTTG TGATCTGACT GCGGAAATGA CCAGCCTCGA TG	pPCRPHB40
BBP2334	NNNTCTAGAG TCACATATGT TTCCCTTCCT TTTTTGTCGG AGACCCTGG	pPCRPHB42
BBP2335	NNNTCTAGAG GATCCAGATC TATGAAAGAG GTTGTAATCG TCGCTG	pPCRPHB42
BBP2802	NNNGGTACCG ATGCACGGCG CTGGACACCT TGTTGAC	pPCRDPHB1
BBP2803	NNNAAGCTTC AGGTTTTCCA GGGTCCTCTG CAGCCAG	pPCRDPHB1
BBP2804	NNNGGATCCC TCAACTGCGC GCCATCCACA AGATGATC	pPCRDPHB2
BBP2805	NNNGGATCCC TTCGGCGCTG GCGACGCTGA GCAGCATC	pPCRDPHB2
BBP2823	NNNTCTAGAC ATATGCGCCC ATTACATCCG ATTGATTTTA TATTC	pPCRWE390
BBP2824	NNNGAATTCT TAATTGGCTG TTTTAATATC TTCCTGCTTT G	pPCRWE390
BBP2951	NNNGAATTCG CAAGCTGTGC GAGCTGCTGC ATCG	pPCRTAGD1
BBP2952	NNNAAGCTTG ATCACCGAGG CCTCGAACGA GGGCAC	pPCRTAGD1
BBP2953	NNNGGATCCT AGGAGGTATT TCCCCGTAAC GCGG	pPCRTAGD2
BBP2954	NNNGGATCCT TTCCCCGTCA ACAAGCCGCC AG	pPCRTAGD2
BBP3002	NNNAAGCTTC TGCCGTTGTG CAGGTGCAC	pPCRALGD3
BBP3022	NNNGGTACCG GATCCGACAT CGAACGGCTC GAAGACATCT AC	pPCRALGD3
BBP3023	NNNAAGCTTG TCAGGATCCG CATACTGCAC CTACATAGCC CAGTCCGAAA ATG	pPCRALGD4
BBP3024	NNNGAATTCG AGCGTCGTTA CTCGTCGTTC GTTTGG	pPCRALGD4
BBP3232	NNNGGATCCG AGCATATGAT CATCCTGCTC TCAAAAAGAT GCGCTTTC	pPCRARS60
BBP3233	NNNGAATTCC ATCGTGCCAA AGCAAATCTA AAGGATTC	pPCRARS60
BBP3234	NNNGGATCCG AAACCGCCGG CGCATCCGGC CGCTGAC	pPCRARS61
BBP3235	NNNTCTAGAC GATGGCGACC CGCTGCTGCT G	pPCRARS61
BBP3407	GTTATTGTTA TTGGCTGTTT TAATATCTTC CTGCTTTGCA ATTACGC	pPCRALC83‐3
BBP3408	AATAACAATG CAATACAGCA GGTACATCAC GCTGACACTT C	pPCRALC83‐3
BBP3948	NNNAAGCTTG AAGCCAATAT CGAAGTGGAC ATGATCGTG	pPCRRSMA1
BBP3949	NNNGAATTCG AAAGTGCCTT CGGAAAACCG GACAGGATTT C	pPCRRSMA1
BBP3950	NNNGGATCCT TTTTCTCAAT TTTTGGCTTT GCAAACGGGG CAAAGGTGG	pPCRRSMA2‐2
BBP3951	NNNGGATCCG AGGGTCTCTC CGACCCGACG AGTCAGAATC AG	pPCRRSMA2‐2
BBP3952	NNNAAGCTTG ATCATCAGGC GCCGCTTCAT G	pPCRFAD1
BBP3953	NNNAGATCTG TTCGATCCGA AAGTCCGAAA GGACTG	pPCRFAD1
BBP3954	NNNGGATCCG AACGAACCCG CGTGCCCGCG CACATC	pPCRFAD4
BBP3955	NNNGGATCCG GGCATCCTCC CGGGTAGGGG CAGAC	pPCRFAD4

### 2.4 Lipid Analysis by GC and GC/MS

Gas chromatography (GC) was used to quantify lipids according to Barney et al. (2012).

### 2.5 Transmission Electron Microscopy

Transmission electron micrographs were obtained through the University Imaging Centers at the University of Minnesota using the JEOL JEM‐1400Plus Transmission Electron Microscope.

### 2.6 Alkylresorcinol Extraction and Purification

**Figure 1:**
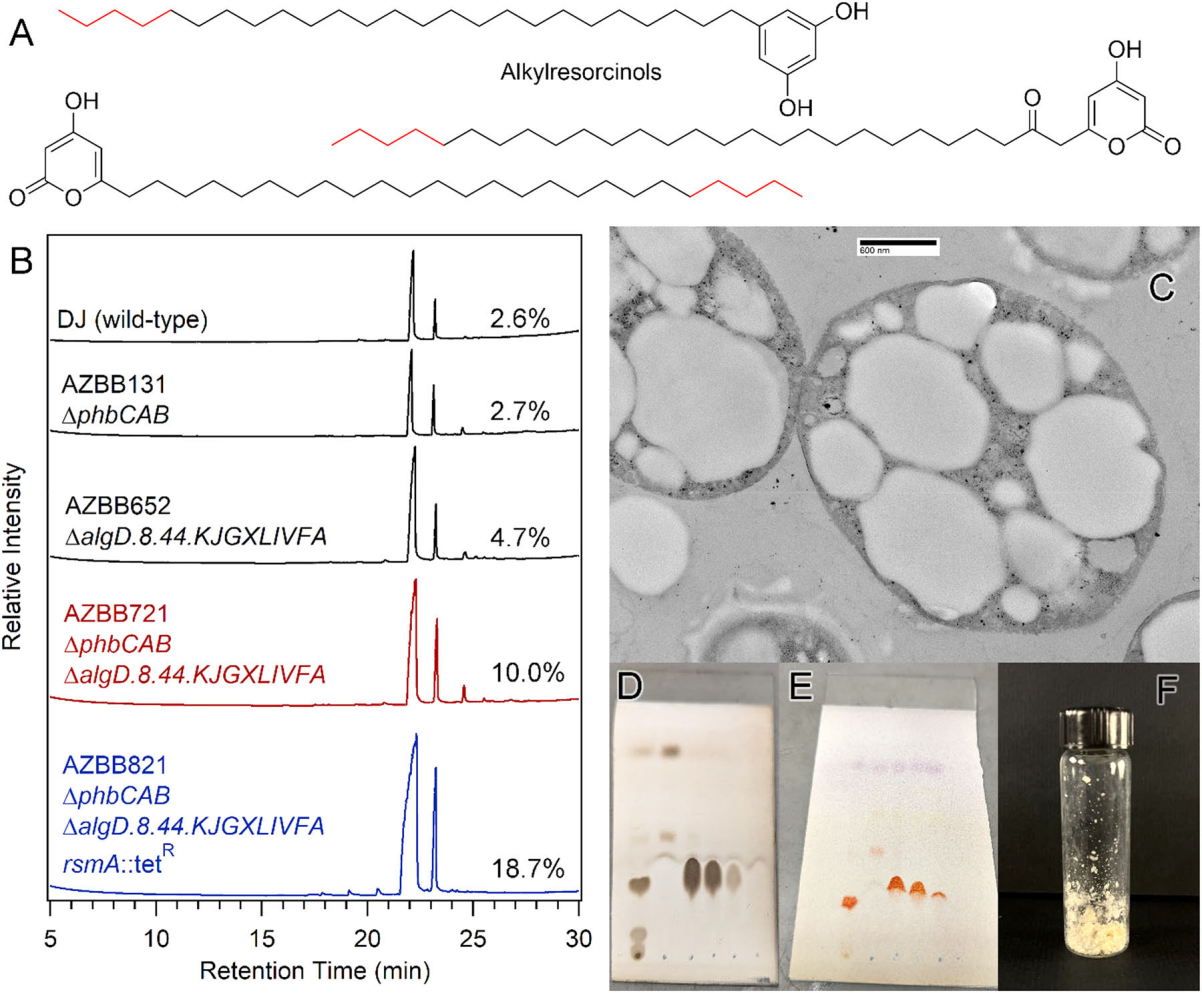
Alkylresorcinol production in *Azotobacter vinelandii*. Shown in panel A are chemical structures for various classes of alkylresorcinols that are produced by *A. vinelandii*. Bonds shown in red are intended to indicate variability in the long chain alkane length of the lipids. Panel B shows representative chromatograms of the lipid fractions obtained from various strains of *A. vinelandii* constructed in this study. The alkylresorcinols correspond to the two peaks at approximately 22 and 23 min. Panel C shows a section from a transmission electron micrograph image of strain AZBB821. The alkylresorcinols manifest as large inclusions that are absent in the parent strain AZBB131. Panels D and E represent thin‐layer chromatography (TLC) plates of the lipid fraction obtained from AZBB821. Lane 1 (left) is the initial fraction, and lanes 3–5 were pooled from a chromatographic separation and the solvent removed to yield the final purified alkylresorcinol shown in panel F. The TLC plate in panel D was charred with sulfuric acid, and the TLC plate in panel E was stained with anisaldehyde.

Cells of *A. vinelandii* strain AZBB821 were grown on standard Burk's medium for 4 days, and were pelleted by centrifugation at 7000 g for 8 min. Supernatant was removed, and the pellet was frozen and then lyophilized to remove any residual water. The lipid fraction was extracted using a mixture of equal parts methylene chloride, *n*‐hexane and tetrahydrofuran. The extract was concentrated in vacuo, redissolved in 3 mL of hexanes, diisopropyl ether, and dichloromethane (equal parts) and separated via flash chromatography on a silica gel column using a mixture of hexanes/diisopropyl ether/acetic acid (50/50/0.04 ratio, respectively). Fractions of 20 mL were collected, and fractions 2–4 (Figure 1) were combined and concentrated in vacuo to yield 0.236 g of the target compound (wax solid, Figure 1F) from 1.2 g of dry cells. This purified fraction of alkylresorcinols was used as a standard for further GC analysis. Thin‐layer chromatography was performed on commercial plates (Analtech) and visualized by charring after spraying with 20% sulfuric acid (Figure 1D) or heating after spraying with anisaldehyde solution (Figure 1E; Stahl and Jork 1965).

## 3 Results

### 3.1 Biosynthesis of Elevated Levels of Native Alkylresorcinols

Native strains of *A. vinelandii* accumulate high concentrations of the extracellular polysaccharide alginate as well as high levels of the intracellular biopolymer PHB (Peña et al. 2002; Segura, Guzmán, et al. 2003). Both of these polymers represent large carbon sinks for *A. vinelandii*, and would be expected to compete for carbon metabolites and energy versus other desired products from biosynthetic schemes. Deletion of genes from either the alginate or the PHB pathways is reported to shift the ratios of the alternative product (Peña et al. 2002; Segura, Guzmán, et al. 2003), indicating that these pathways interact and compete with one another for intracellular resources. Additionally, under certain conditions, *A. vinelandii* undergoes a metamorphosis to form desiccation‐resistant cysts (Stevenson and Socolofsky 1966). During the formation of the cysts, cells accumulate elevated quantities of PHB and an additional lipid referred to as alkylresorcinol (Stevenson and Socolofsky 1966; Segura, Cruz, et al. 2003). Various regulatory networks are involved in managing the production of these different compounds, and the alkylresorcinols that are produced begin to replace phospholipids in the membranes during cyst production (Segura, Cruz, et al. 2003; Cocotl‐Yañez et al. 2011; Muriel‐Millan et al. 2015; Romero et al. 2013; Segura et al. 2009). The alkylresorcinol is a highly reduced waxy compound with the potential to serve as a feedstock to produce other reduced forms of carbon, and can be enhanced by growing *A. vinelandii* vegetative cells on *n*‐butanol or β‐hydroxybutyrate (Segura, Cruz, et al. 2003).

Because we believed that alkylresorcinol, PHB, and alginate accumulation would compete with other neutral lipids that we aimed to produce in *A. vinelandii*, we first directed our efforts to construct strains that removed key genes involved in the biosynthesis of these alternative products. For these various strategies, we employed genome engineering strategies that allow us to make extensive modifications to the *A. vinelandii* genome without leaving behind antibiotic markers. Our laboratory has developed two separate approaches for making markerless modifications in *A. vinelandii* that utilize either indigenous *pyrF* (Eberhart et al. 2016) or foreign *sacB* (Dietz et al. 2024) genes for conditional toxicity approaches, followed by counterselection to remove antibiotic resistance genes in a markerless manner. The use of these systems enabled the extensive genome modifications that we describe here.

As a first step, we targeted the *phbBAC* operon (AZBB131) or the large alginate *algD.8.44.KJGXLIVFA* operon (AZBB652) for deletion of PHB and alginate, respectively. Following independent removal of each of these larger clusters of genes, we observed a subtle increase in alkylresorcinol for AZBB652 (Figure 1B). A further strain was constructed that combined the deletion of both of these operons into one single strain (AZBB721), which resulted in further accumulation of alkylresorcinol. Hernandez‐Eligio et al have previously reported that RsmA posttranscriptionally represses the expression of PhbR (Hernandez‐Eligio et al. 2012). On the basis of this prior report, we speculated that RsmA may also act as a global regulator in *A. vinelandii* that is also hindering resorcinol production. To test this hypothesis, we disrupted the *rsmA* gene in an additive manner, generating the extensively modified strain AZBB821, which resulted in further accumulation of alkylresorcinol, achieving concentrations approaching 20% of the total dry mass of the cell, as determined by gas chromatographic and gravimetric analysis (Figures 1B and 1F). Further analysis of cellular composition using scanning transmission electron microscopy revealed large inclusions of alkylresorcinols that were not present in the preceding AZBB131 strain (Barney 2024), indicating that these lipids accumulate in the cytoplasm (Figure 1C).

While strain AZBB721 grew at a similar rate to the wild‐type (DJ) strain (Knutson et al. 2018), the addition of the disruption to *rsmA* did incur a growth penalty, resulting in a decrease in the doubling time from about 3 h under diazotrophic growth to about 7 h for AZBB821, likely due to the high accumulation of intracellular lipids, indicating that this increase in metabolic redistribution did result in some degree of cellular stress.

### 3.2 Biosynthesis of Wax Esters in *A. vinelandii*

**Figure 2:**
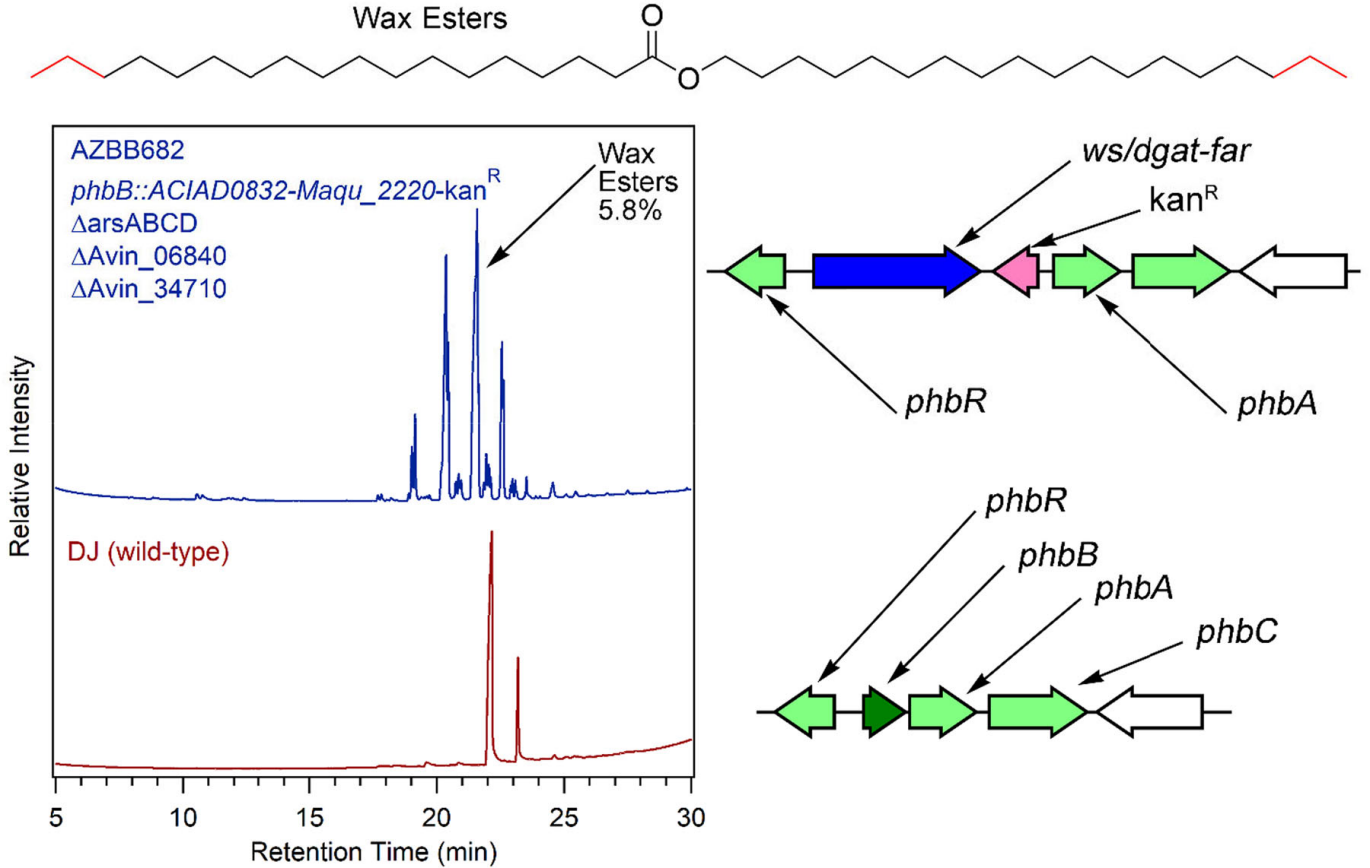
Wax ester production in *Azotobacter vinelandii*. Shown above are chromatograms for strain AZBB682 and wild‐type *A. vinelandii*. Wild‐type *A. vinelandii* accumulates resorcinol, which appears as two sharp peaks at approximately 22 and 23 min. Strain AZBB682 lacks the *arsABCD* alkylresorcinol operon, the triglyceride lipase *Avin_06840*, and the PHB depolymerase *Avin_34710*. It also carries the foreign *ws/dgat* from *A. baylii* and the Maqu_2220 gene encoding a fatty acyl‐ACP/fatty aldehyde reductase (*far*) from *Marinobacter aquaeolei* VT8 fused as a single protein product, and accumulates wax esters shown as a series of peaks between 19 and 23 min. Bonds shown in red are intended to indicate variability in the carbon length of the fatty alcohols and fatty acids of the wax esters (top). Shown on the top right is an illustration of how the *ws/dgat‐far* gene fusion has been targeted to the *phbB* promoter in *A. vinelandii*, versus the wild‐type arrangement of the *phbBAC* operon.

To reconfigure intracellular wax accumulation in *A. vinelandii* for the production of wax esters, we constructed a strain without the *arsABCD* operon, the triacylglyceride esterase *Avin_06840* or the PHB depolymerase *Avin_34710* (also a potential esterase). The two esterases were removed as a precautionary measure. The strain AZBB682 was constructed by inserting the *ws/dgat* gene from *Acinetobacter baylii* fused to the *Maqu_2220 far* gene from *M. aquaeolei* VT8 (recently renamed *M. nauticus*) behind the *phbB* promoter of *A. vinelandii*. This construct resulted in the accumulation of wax esters (Figure 2) that were not present in the wild‐type DJ strain. This profile of lipids is similar to the wax ester profile observed for *M. aquaeolei* VT8 and *A. baylii* (Wältermann et al. 2007; Barney et al. 2012), confirming that the incorporation of the fused gene product into the genome of *A. vinelandii* is sufficient for the accumulation of wax esters. Quantification by GC indicated that the levels accumulated accounted for nearly 6% of the dry weight of the cell material. As expected, no accumulation of alkylresorcinol was observed in this construct.

### 3.3 Biosynthesis of Fatty Alcohols

**Figure 3:**
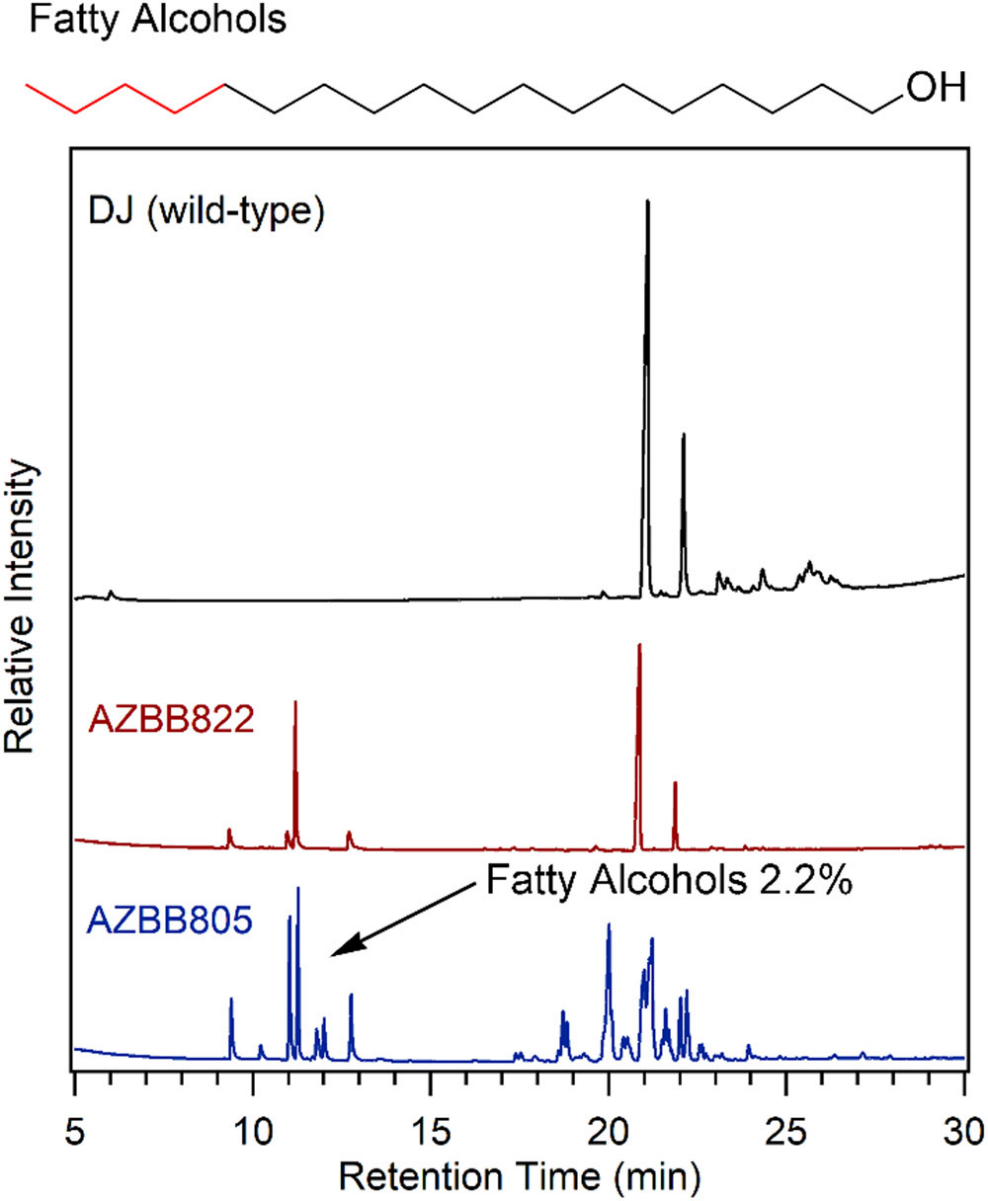
Fatty alcohol production in *Azotobacter vinelandii*. Shown above are chromatograms for various strains of *A. vinelandii*. Wild‐type *A. vinelandii* accumulates resorcinol, which appears as two sharp peaks at approximately 21 and 22 min. Strain AZBB822, which contains the Maqu_2220 gene encoding a fatty acyl‐ACP/fatty aldehyde reductase along with a disruption in the Avin_15490 (fatty acid degradation pathway) accumulates a variety of C14‐C18 fatty alcohols eluting between 9 and 13 min. Strain AZBB805, which is identical to AZBB822, but also contains the *ws/dgat* gene from *Acinetobacter baylyi* produces wax esters in place of alkylresorcinol along with elevated levels of fatty alcohols. Bonds shown in red are intended to indicate variability in the carbon length of the fatty alcohols (top).

*A. vinelandii* does not contain a known fatty acyl‐CoA reductase or fatty acyl‐ACP reductase. As a result, it does not naturally accumulate fatty alcohols. In the process of constructing strains to determine if disruptions in key genes of the fatty acid degradation pathway would impact wax ester accumulation, we observed that disruption of *Avin_15490* resulted in the accumulation of fatty alcohols in *A. vinelandii*. The *Avin_15490* gene encodes an acyl‐CoA dehydrogenase that plays an important role in fatty acid degradation. Addition of disruptions of this gene to strains already containing the *ws/dgat*‐*far* fusion gene in addition to PHB and alginate disruptions (AZBB744) resulted strain AZBB805, which accumulated wax esters, but also accumulated an apparent excess of fatty alcohols. Quantification of the fatty alcohols by GC indicated that these free fatty alcohols accounted for just over 2% of the cell dry mass. Interestingly, the accumulation of wax esters was accompanied by a significant decrease in the accumulation of alkylresorcinols versus the AZBB721 strain (Figure 3). As a further test, we constructed strain AZBB822, which contains the PHB and alginate disruptions of AZBB721, but also the *Avin_15490* gene disruption for fatty acid degradation (AZBB808). Further, instead of the fused *ws/dgat*‐*far* gene fusion, we only incorporated the *far* gene (*Maqu_2220*) alone in AZBB822, resulting in a strain that accumulated fatty alcohols, but not wax esters. In AZBB822, we still observed some resorcinol accumulation, indicating that the fatty alcohols are not hindering alkylresorcinol yields in the same manner that wax esters do (Figure 3). This interdependency between the three classes of neutral lipids is an interesting and unexpected caveat of our strategies that will require more complex strain construction in the future to determine possible explanations for these differences.

## 4 Discussion

There are many examples in the literature of efforts to produce various oils and waxes in microbial hosts as a potential replacement for petroleum hydrocarbons (Steen et al. 2010; Wältermann et al. 2005; Pfleger et al. 2015; Schirmer et al. 2010). Most of these approaches use common laboratory strains of yeasts or bacteria such as *E. coli* as a host for these biosynthetic routes. In most of these cases, the production of the hydrocarbon from these hosts would require feedstocks that contain a source of fixed nitrogen as a component of the growth medium. *A. vinelandii* is a common laboratory microbe that can be grown on a very simple medium composed primarily of sugars or other carbohydrates, and is able to obtain all of its nitrogen requirements from the process of biological nitrogen fixation through the enzyme nitrogenase (Barney 2024; Setubal et al. 2009). In this manner, *A. vinelandii* is nitrogen negative, resulting in the accumulation of fixed nitrogen in the remaining biomass that does not need to be derived from industrial nitrogen fixation processes. Our laboratory has pursued efforts over the past decade to yield elevated quantities of extracellular nitrogen compounds from cultures of *A. vinelandii*, demonstrating high yields of both ammonium and urea (Barney, Eberhart, et al. 2015; Plunkett et al. 2020; Barney and Dietz 2024; Barney and Plunkett 2022). In a recent report, we also demonstrated that modified strains of *A. vinelandii* still accumulate high levels of PHB in addition to the extracellular nitrogen that can be obtained (Barney and Dietz 2024). In this manner, it is possible to culture *A. vinelandii* and obtain elevated levels of ammonium from the spent medium, and then extract the PHB from the cell mass as a source of potential bioplastics.

Our laboratory also studies the enzymes associated with biological wax production based on the biosynthesis of wax esters, a biological wax that had been historically harvested from the whaling industry (Barney et al. 2012, 2013; Barney, Ohlert, et al. 2015; Knutson et al. 2017; Lenneman et al. 2013; Stöveken et al. 2005; Wahlen et al. 2009; Willis et al. 2011). These wax esters are a common component of the outer waxes that protect plant tissues and that are also found on the outer dermal layer of our own skin (Busta and Jetter 2018; Jetter and Kunst 2008). In more recent years, alternative biological sources such as jojoba plants have replaced the prior whaling source, and there is interest in applying other strategies to plants and microbes for the production of similar waxes (Kalscheuer et al. 2006; Metz et al. 2000; Miwa 1971). For these reasons, we were interested in investigating the potential to produce wax esters and other natural lipid hydrocarbons in *A. vinelandii*.

Our initial attempts to produce wax esters in *A. vinelandii* revealed levels of alkylresorcinols in the parent laboratory DJ strain that represented a significant quantity of material that could interfere in this effort. Additionally, we recognized that PHB might also compete for intracellular resources in any attempts to accumulate lipids. In prior work, we had recognized that while the DJ parent strain is diminished in alginate production, there are still significant levels of alginate in the growth medium, even though the goopy phenotype associated with the native *A. vinelandii* strain is seemingly disrupted (Barney 2024). The DJ strain contains a simple gene disruption in the *algU* gene, however, alginate production in *A. vinelandii* is based on a large suite of genes that are all present in *A. vinelandii* (Setubal et al. 2009). For these reasons, we set about the task of constructing strains that were devoid of these competing pathways to establish an initial parent strain that would be more amenable to lipid accumulation. During the construction of these strain, we realized that various combinations of these gene disruptions resulted in increased accumulation of alkylresorcinol. A combination of PHB, alginate, and *rsmA* disruptions resulted in the highest increase in lipid accumulation, representing a sevenfold increase in this natural lipid that is generally associated with cyst production. Contrary to the general narrative that alkylresorcinols are primarily associated with the outer membrane (Stevenson and Socolofsky 1966; Segura, Cruz, et al. 2003), in this study, we found the alkylresorcinols accumulating as intracellular inclusions in the cytoplasm (Figure 1C). These results indicate that as an alternative to PHB, *A. vinelandii* can be reconfigured to produce high levels of a suitable native biofuel precursor that can be easily extracted from dried cell mass, using simple solvents without the requirement for fixed nitrogen compounds in the growth medium.

In addition to alkylresorcinols, we also reprogrammed *A. vinelandii* to produce wax esters. A simple disruption to the *Avin_15490* gene that is instrumental in fatty acid degradation resulted in the accumulation of fatty alcohols in addition to wax esters, indicating that *A. vinelandii* could be a suitable substitute to more common laboratory strains, such as *E. coli*, which can serve as a chassis for the production of biofuels, but is dependent on the provision of fixed nitrogen compounds in the growth medium to support its growth. While wax esters have potential applications as high‐value lubricants and components of certain cosmetics and skincare production, fatty alcohols could serve as a drop in replacement for diesel fuels. Our results support the potential to produce a range of different biological neutral lipids either alone or as part of a more complex lipid matrix that could serve as a potential crude oil replacement.

## 5 Conclusions

In this study, we have constructed a series of *A. vinelandii* strains with simple gene disruptions that accumulate high levels of a native biological wax while preserving the ability to fix atmospheric nitrogen under aerobic conditions, a hallmark of the *A. vinelandii* strain. These results illustrate the potential of *A. vinelandii* to serve as a model chassis for the production of biofuel‐related compounds in a manner that is nitrogen negative. When coupled together with prior modifications, we obtained yields of nearly 20% of the cell dry mass as alkylresorcinols. We were further able to demonstrate the production of both wax esters and fatty alcohols through the heterologous expression of foreign genes from wax ester‐accumulating hosts. These results illustrate the potential of *A. vinelandii* to serve as a suitable chassis microbe for the production of bio‐oils and bio‐waxes without the requirement for reduced nitrogen compounds in the culture medium.

## Author Contributions

**Brett M. Barney:** conceptualization, methodology, investigation, validation, visualization, funding acquisition, writing – original draft, writing – review and editing, project administration, formal analysis, supervision. **Bilge Bahar Camur:** methodology, investigation, formal analysis, writing – review and editing. **Lucas J. Stolp:** methodology, investigation, formal analysis, writing – review and editing. **Natalia Calixto Mancipe:** methodology, investigation, formal analysis, writing – review and editing. **Benjamin R. Dietz:** methodology, writing – review and editing, investigation, formal analysis.

## Ethics Statement

The authors have nothing to report.

## Conflicts of Interest

B.M.B. has applied for provisional patents related to the hyper accumulation of alkylresorcinol in *A. vinelandii*.

## Data Availability

The data that support the findings of this study are available from the corresponding author upon reasonable request. All data generated or analyzed during this study are included in this published article.
